# Age structure and parity status determination of Afrotropical malaria vectors using MALDI-TOF MS

**DOI:** 10.1038/s41598-025-00935-1

**Published:** 2025-05-06

**Authors:** Mercy Tuwei, Jonathan Karisa, Caroline Kiuru, Zedekiah Ondieki, Tobias Odongo, Martha Muturi, Festus Mure, Bruno Otieno, Miguel Okoko, Brian Bartilol, Rehema Gona, Kelly Ominde, Luis Constantino, Gildo Cole, Trisa Anastácio, Baltazar Candrinho, Romário Armazia, Claudia Alves, Edith Ramaita, Martin Rono, Joseph Mwangangi, Charles Mbogo, Francisco Saute, Carlos Chaccour, Caroline Wanjiku, Marta Maia

**Affiliations:** 1 Kenya Medical Research Institute, Wellcome Trust Research Program, Kilifi, Kenya; 2 KEMRI-CGMR Coast, Kilifi, Kenya; 3 National Malaria Control Program, Ministry of Health, Nairobi, Kenya; 4 Centro de Investigação em Saúde de Manhiça, Bairro Cambeve. Rua 12, Distrito da Manhiça, 1929 Maputo, Mozambique; 5 ISGlobal, Carrer Roselló 132, 08036 Barcelona, Spain; 6 CIBER de Enfermedades Infecciosas, 28029 Madrid, Spain; 7 Navarra Center for International Development, Universidad de Navarra, 31009 Pamplona, Spain; 8 National Malaria Control Program, Maputo, Mozambique; 9 Nuffield Department of Medicine, Centre for Global Health and Tropical Medicine, University of Oxford, Oxford, UK

**Keywords:** *Anopheles gambiae s.s.*, *An. funestus s.s.*, Age-grading, MALDI-TOF MS, Parity, Detinova, Biological techniques, Medical research

## Abstract

The age structure of a mosquito population helps estimate the proportion of vectors capable of transmitting malaria. Many malaria transmission models rely on mosquito longevity as key parameter. However, these are rarely measured in the field due to lack of a reliable and scalable age-grading method. An accurate method could improve predictions of malaria risk and the impact assessment of interventions. This study aimed to investigate the use of Matrix-Assisted Laser Desorption/Ionization Time-of-Flight Mass Spectrometry (MALDI-TOF MS) for malaria vector age-grading using insectary-reared and wild-caught mosquitoes. *Anopheles gambiae s.s.* mosquitoes were reared in the insectary to different known physiological and chronological ages to evaluate if MALDI-TOF MS could be used to distinguish between different age groups. Wild mosquitoes were collected from Mozambique and Kenya and dissected to determine their parity status. Reference spectra were obtained from mosquito’s cephalothorax and used to create predictive databases which were validated using independent samples. MALDI-TOF MS identified the physiological and chronological age of insectary-reared mosquitoes with 94.52% and 77% accuracy respectively. Field-collected mosquitoes were primarily *An. funestus s.s.* and *An. gambiae* s.s. Parity prediction accuracy was between 81% and 87%. MALDI-TOF MS was able to distinguish and differentiate mosquitoes based on their age structure (chronological and physiological) and parity status.

## Introduction

For a mosquito to transmit malaria it must feed on humans twice - once to acquire the parasite and a second time to transmit it, with a parasite development phase of at least ten days in between. The ability of a vector to survive long enough to complete the parasite extrinsic incubation period is the most important factor determining vectorial capacity^1^. By tracking changes in the age composition of mosquito populations we can determine the impact of control interventions on mosquito survival rates^2^. Several novel interventions are being developed to reduce vectorial capacity by decreasing mosquito survival. These include endectocides, attractive-targeted sugar baits, spatial repellents, and genetically modified (GM) mosquitoes^3,4^. The age structure of mosquito populations is the most effective indicator for assessing the impact of these control interventions^2^. However, accurately establishing age structure in field populations remains technically challenging and is therefore rarely done at scale.

Traditionally, mosquito age is estimated through ovarian dissections which help provide clues about the reproductive life history of a female. Two techniques are used: the Detinova^5^ and Polodova^6^ methods. The Detinova approach, provides a binary assessment of parity by microscopically observing the ovarian tracheole skeins and classifying mosquitoes as either parous – a mosquito that has laid eggs, or nulliparous – a mosquito that has never laid eggs^5^. The Polovodova method is used to determine the number of oviposition events by distending the ovariole follicular tube and counting the dilations -each indicating completion of a gonotrophic cycle (3–4 days). Both approaches require precise dissection and skilful observation to accurately assess the condition of the reproductive organs which is laborious, time-consuming, and subjective. The Detinova technique is more widely employed than Polodova, perhaps because it is comparably easier. However, it cannot distinguish between more than one egg-laying event, and mosquitoes in pregravid state are routinely classified as nulliparous although they may already be over 7 days old and infectious^7^. A further major limitation of ovarian dissections is that ideally, they need to be conducted on freshly caught specimens, which limits the ability for scale-up.

Ageing mosquitoes induces molecular and biochemical changes, which have been leveraged to develop age-grading assays^8–11^. Techniques such as near infrared spectroscopy (NIRS)^12–14^,have shown some promise although in some studies it was unable to predict the age of wild mosquitoes^14^. Mid-infrared spectroscopy (MIRS)^15,16^ has also been proposed but not without limitations^17^. Generally, spectroscopy approaches use machine learning to collect reference information from samples of known age for the development of training datasets that are then used to predict the age of independent samples. So far, promising results have only been attained using insectary-reared or semi-field reared mosquitos. However, predictive accuracy typically declines when models are applied to wild-caught mosquitoes or to samples from geographic regions different from those used to generate the training data. This performance drop is likely because the training datasets may not adequately capture the biochemical diversity present in wild mosquito populations, limiting the generalizability of age prediction models across settings.

Matrix-assisted laser desorption ionization–time-of-flight mass spectrometry (MALDI-TOF MS) is a diagnostic method used for the identification of microbes (bacteria, fungi) based on the mass-charge ratio (m/z) of peptides or protein molecules. The technology has revolutionized medical microbiology due to its high accuracy levels, robustness^18^, short turn-around time from sample preparation, result generation, and cost-effectiveness compared to classical methods. MALDI-TOF MS is inherently more robust profiling tool than MIRS and NIRS because it targets and analyzes the protein composition of the sample—mainly abundant, stable, and conserved proteins like ribosomal proteins. The protein profiles are highly specific and relatively stable, making them ideal for building reference libraries and for consistent identification across diverse sample sets. On the other hand, MIRS (Mid-Infrared Spectroscopy) captures a broader chemical fingerprint, including proteins, lipids, carbohydrates, and other molecular components. While this holistic approach can be informative, it also introduces greater variability and noise into the data. Factors such as environmental conditions, strain, and seasons can influence MIRS and NIRS spectra more significantly than they do protein profiles in MALDI-TOF MS.

Over the past decade, the technology has increasingly been explored in the entomology field for the identification of different arthropod species such as ticks^19^, biting midges^20^, sandflies^21^, mosquitoes^22^ and including sibling species of the *An. gambiae* and *An. funestus* complexes^23^. The tool has also been applied in the detection of arthropod-borne pathogens such as rickettsia in ticks^24^, filarioid helminths in *Aedes aegypti*^25^ and *Plasmodium berghei* in *An. stephensi*^26^. Additionally, MALDI-TOF MS has been used for the identification of blood meal sources from different hosts^23,27^.

In the case of mosquito age grading, we hypothesize it is possible to develop predictive databases exploiting protein changes that occur due to senescence and natural physiological progression (mating, blood feeding, oviposition) to determine whether the mosquito is young or old. Several studies have readily shown this potential using insectary-reared *Anopheles stephensi* and field-caught *Anopheles arabiensis* reared to different ages in controlled conditions^28,29^. These studies employed machine learning methods such as convolutional neural networks (CNN) and artificial neural networks (ANN) however their training and testing datasets were mosquitoes that had undergone the ageing process in controlled insectary conditions.

In this study, we employed MALDI-TOF MS to analyze protein profiles from insectary-reared mosquitoes of known chronological and physiological ages. Our goal was to determine whether the protein signatures used in our predictive models are primarily influenced by senescence—the natural aging process measured in days—or by key physiological events in the mosquito lifecycle, such as mating, blood feeding, and oviposition. Importantly, this is the first study to successfully predict the parity status (i.e., nulliparous versus parous) of wild-caught Afro-tropical mosquito vectors that aged under natural environmental conditions.

## Results

### Phase I results – insectary reared *Anopheles gambiae* s.s.

#### Physiological age prediction

**Fig. 1:**
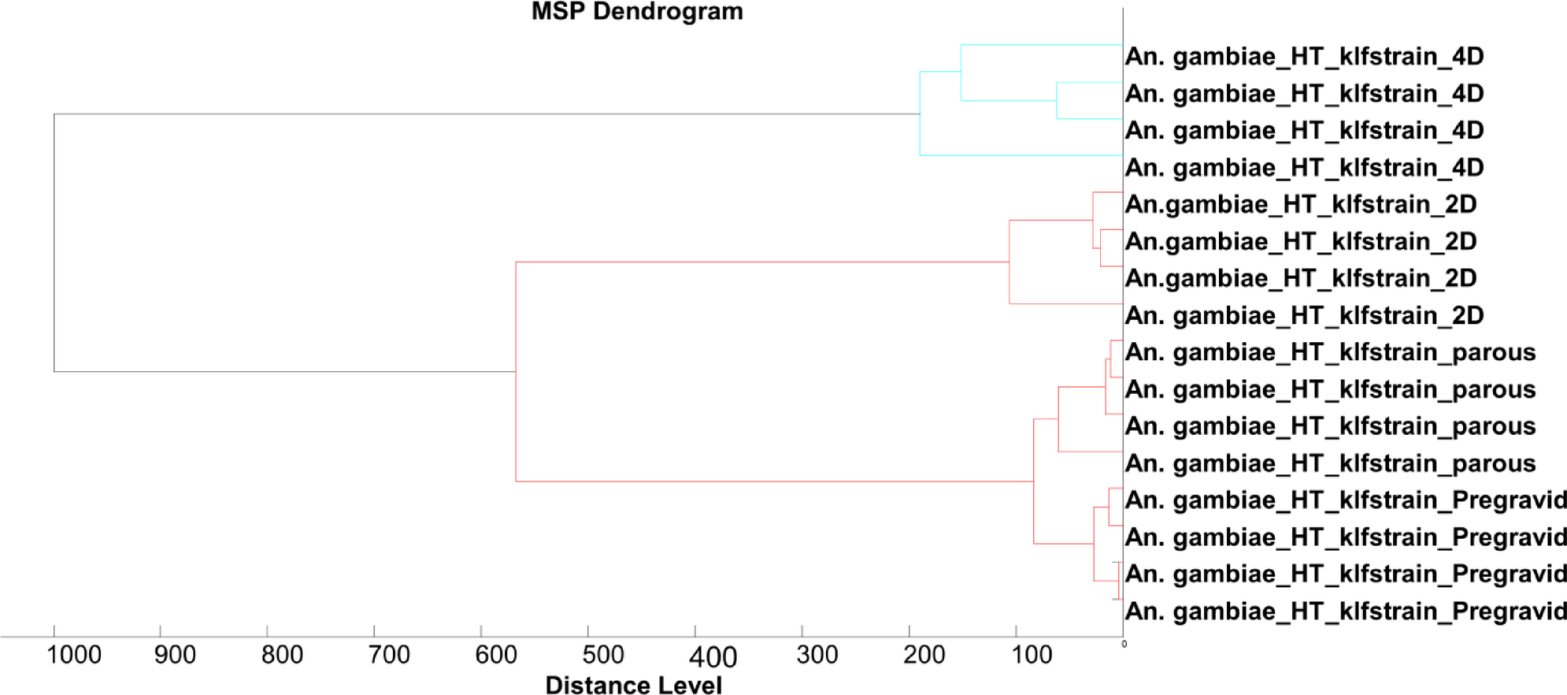
MSP dendrogram of the spectra used for database creation, and categorized by physiological status.

MALDI-TOF MS spectra were obtained from a total of 356 *An. gambiae s.s*. Sixteen samples were excluded from further analysis due to poor quality spectra. From the remaining spectra (340 samples), 30 spectra were used for database creation (training dataset) and 310 for database query to measure the performance of the database (test dataset). A dendrogram generated using a subsample of the training dataset showed clear separation of spectra into three different groups: (i) 2-days, (ii) 4-days and (iii) pregravid and parous) (Fig. 1) with log score values exceeding 1.8 (Fig. 3). Despite the parous and pregravid clustering together, they occupied different branches, clearly indicating specificity even for closely related categories (Fig. 1). The overall accuracy for categorization was 94.5%, and 97% (2-days old), 89% (4-days old), 100% (pre-gravid) and 97% (parous) for the respective groups (Table 1).

**Table 1:** Performance of MALDI­TOF MS in predicting the physiological age of insectary reared mosquitoes.

MALDI-TOF MS age prediction	Reference			
	2 days	4 days	Pregravid	Parous
2 days	53	6	0	0
4 days	2	56	0	0
Pregravid	0	0	41	0
Parous	0	9	0	143
Sensitivity	96.4	78.9	100	100
Specificity	97.7	99.2	100	94.6
Accuracy	97	89	100	97.3
Overall accuracy	94.52			
Kappa	0.9193			

#### Chronological age prediction

**Fig. 2:**
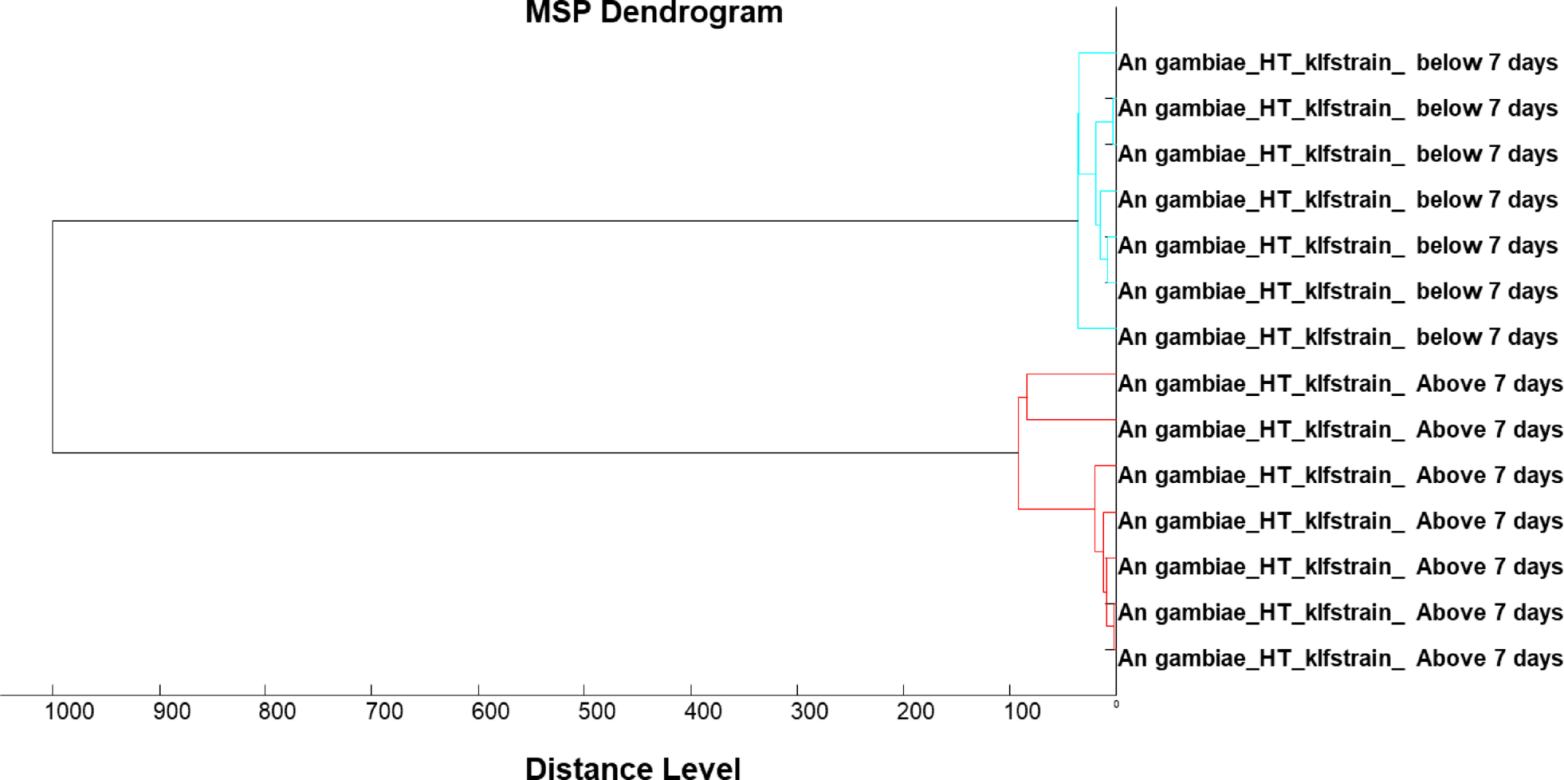
MSP dendrogram of the spectra used for database creation for chronological age categorization.

**Fig. 3:**
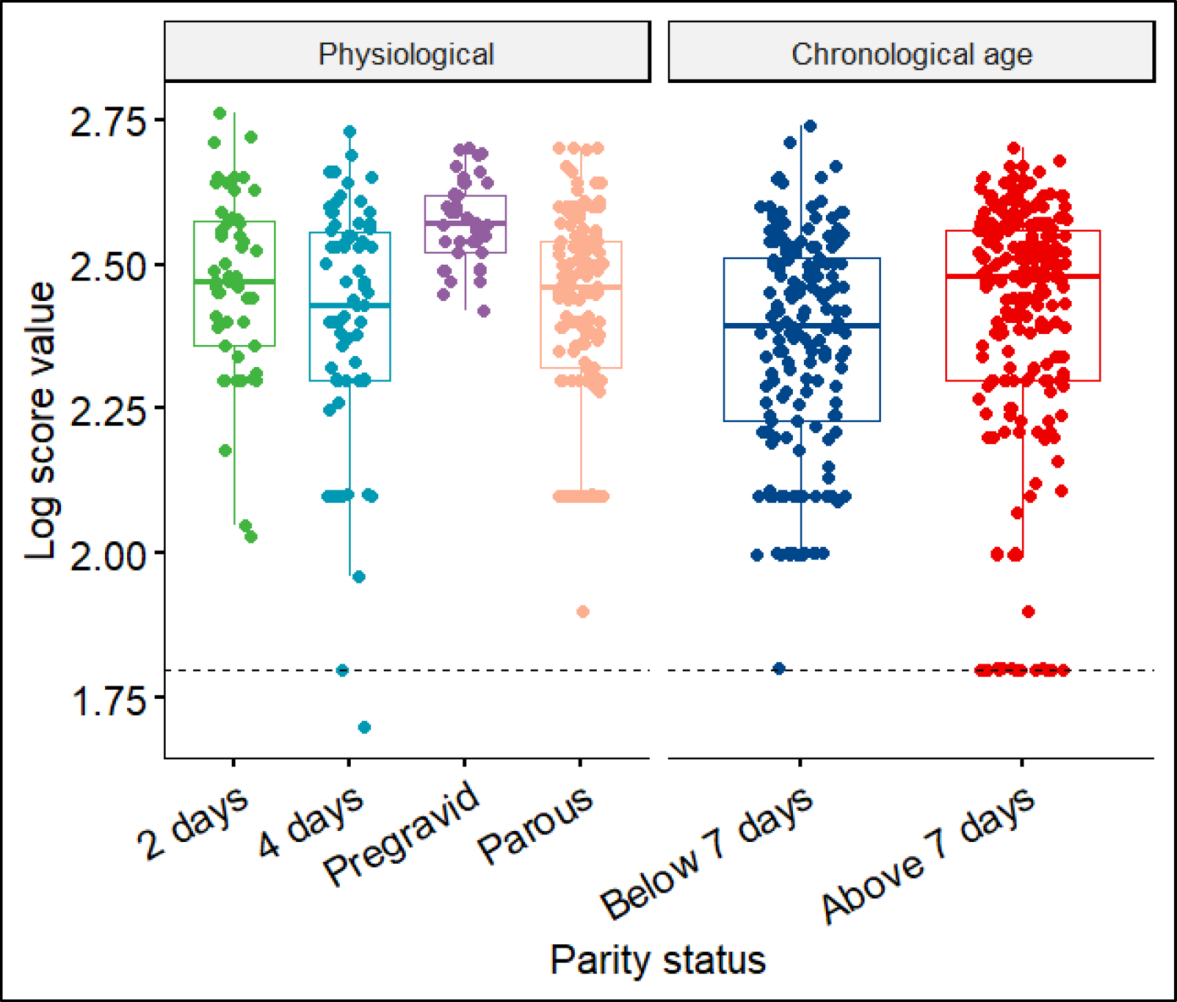
Box plot showing the log score values (LSVs) of all the spectra queried against physiological and chronological age databases.

A total of 442 MALDI-TOF MS spectra were obtained,14 excluded due to poor spectra quality, 33 used for database creation (training dataset), 395 for database query (test dataset). Two age categories were considered as epidemiologically relevant: (i) below 7 days old; and (ii) 7 days old and above. The overall accuracy for chronological age categorization was 77% (Table 2). A dendrogram generated using training dataset showed a clear separation between younger (< 7days old) and older mosquitoes (> 7days) (Fig. 2) and with log score values of above 1.8 (Fig. 3).

**Table 2:** Performance of MALDI­TOF MS in predicting the chronological age of insectary reared mosquitoes.

Species	Site and storage conditions	Total number	Chronological age		MALDI- TOF MS predictions		Specificity (95% CI)	Sensitivity (95% CI)	Accuracy (95% CI)	Kappa (95% CI)
			< 7	> 7	< 7	> 7				
*An. gambiae* s.s.	Fresh samples	395	146	249	164	231	66(59–74)	84(79–88)	77(72–81)	0.51(0.43–0.60)

### Phase II results – Parity prediction of wild-caught anophelines from Mozambique and Kenya

A total of 810 MALDI-TOF M spectra were obtained from mosquitoes caught in Mozambique (*An funestus s.s* (*n* = 500); *An. gambiae* s.s. (*n* = 310)). 87% (436/500) of the *An. funestus s.s*, were parous and 12.8% (64/500) nulliparous. For *Anopheles gambiae s.s* 91.2% (283/310) were parous and 8.7% (27 /310) nulliparous (Table 3).

**Table 3:** Performance of MALDI-­TOF MS in predicting the age of wild collected mosquitoes.

Species	Site and storage conditions	Total number	Parity dissections^1^		MALDI- TOF MS predictions		Specificity (95% CI)	Sensitivity (95% CI)	Accuracy (95% CI)	Kappa (95% CI)
			True NP	True *P*	Pred NP	Pred *P*				
*An. funestus* s.s.	RNA later	500	64	436	144	356	77(64–86)	78(74–82)	78(74–82)	0.36(0.27–0.45)
*An. gambiae* s.s.	RNA later	310	27	283	39	271	48(29–68)	91(87–94)	87(83–91)	0.32(0.17–0.48
Overall	RNA later	810	91	719	183	627	68(58–78)	83(80–86)	81(79–84)	0.36(0.28–0.43)
*An. funestus* s.s.	Kwale - Ke (frozen)	332	104	228	100	232	77(68–85)	91(87–95)	87(83–90)	0.69(0.6–0.77)

From the mosquitoes collected in coastal Kenya a total of 332 MALDI-TOF MS spectra were obtained. These were composed uniformly of An. *funestus* s.s. (332) of which 228 were parous and 104 were nulliparous (Table 3).

#### Spectral analysis and database creation for parity status determination

**Fig. 4:**
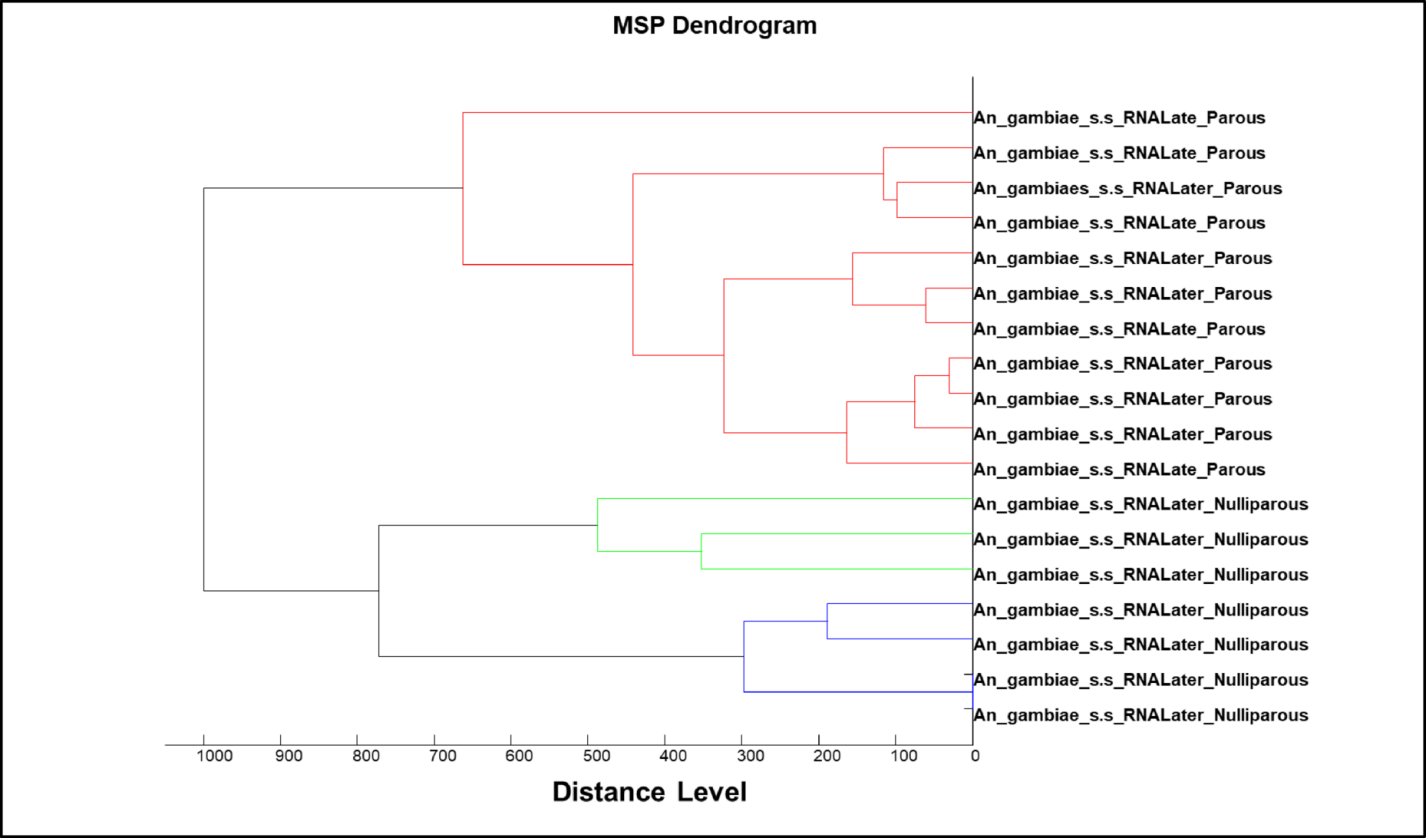
MSP dendrogram of spectra from wild collected *An. gambiae* s.s stored in RNALater and used for database creation.

**Fig. 5:**
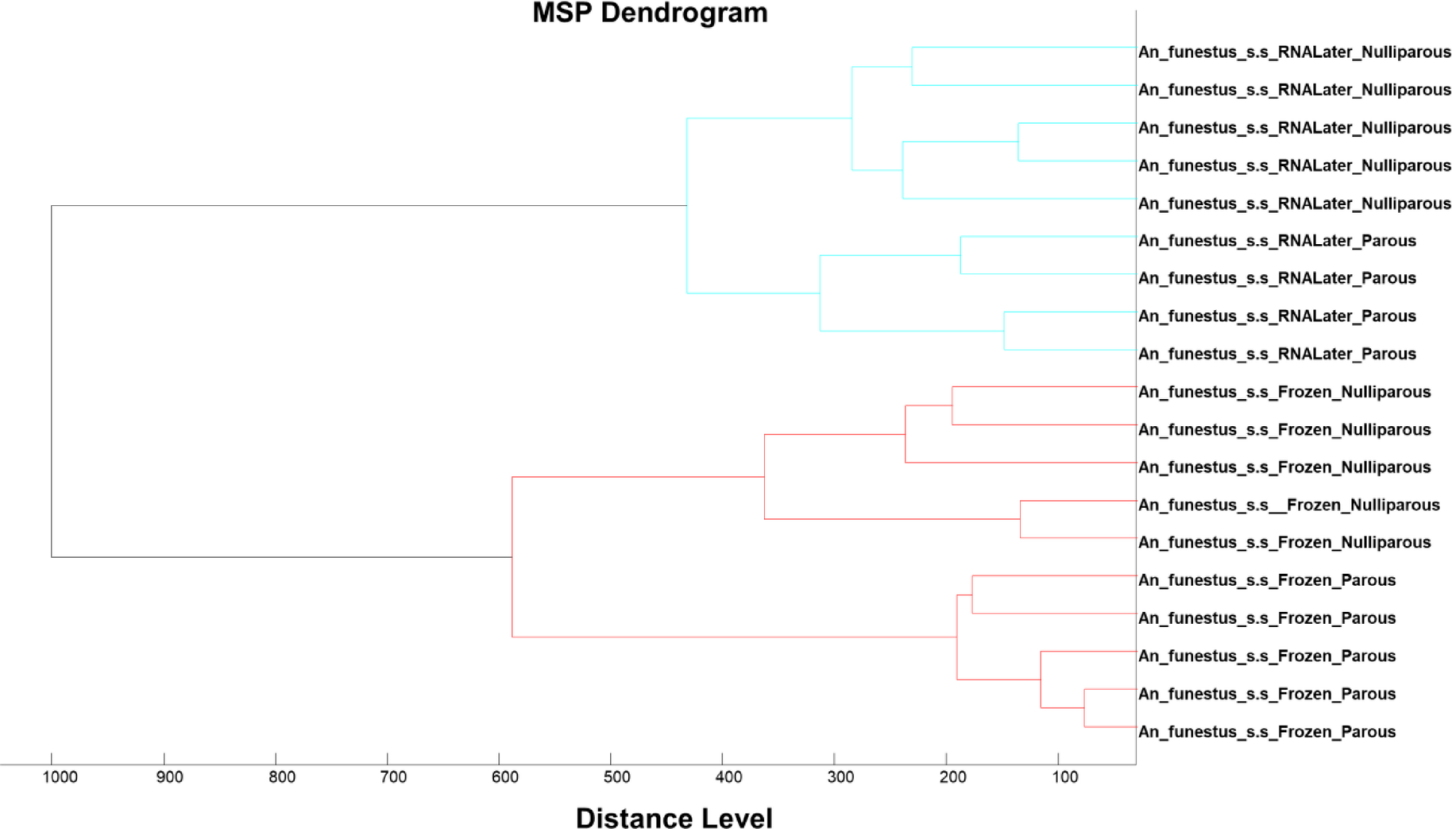
MSP dendrogram of spectra obtained from wild collected *An. funestus* s.s. stored in RNAlater and frozen (-20 C) and used for database creation.

Overall, 1142 wild mosquito samples were used for parity analysis, composed of *An. funestus* s.s (664 parous; 168 nulliparous) and *An. gambiae* s.s. (283 parous; 27 nulliparous) (Table 3). Thirty-seven mosquitoes were used for database creation. *An. arabiensis* and *An. rivulorum* were not included in the database as they were in insufficient number. A dendrogram generated using the 37 spectra of *An. gambiae* s.s. (18, 7 nulliparous and 11 parous). and *An. funestus* s.s (19, 10 nulliparous and 9 parous) were used for database creation, revealing distinct separation by parity status (Fig. 4). In the case of *Anopheles funestus*, the dendrogram also showed a clear separation between parous and nulliparous mosquitoes for each type of storage condition (Fig. 5).

#### Impact of sample storage conditions on performance of MALDI-TOF MS

**Fig. 6:**
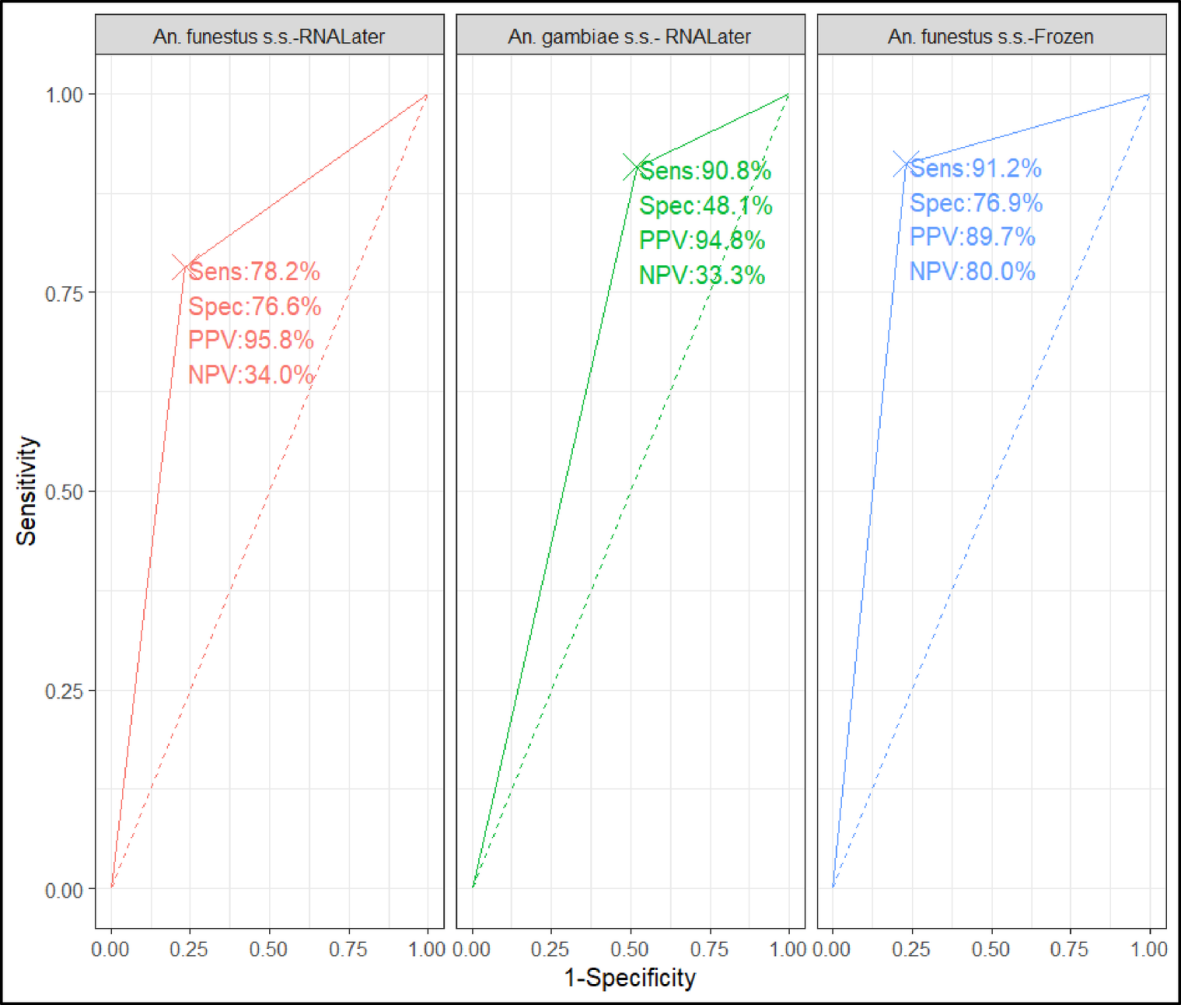
The ROC curve visually represents the diagnostic capability of a binary classifier system across different discrimination thresholds. It includes three variations: the ROC curve specifically for Mozambique samples (*An. funestus s.s.* and *An. gambiae* s.s. stored in RNALater) and for Kenya samples *An. gambiae* s.s. which were frozen.

**Fig. 7:**
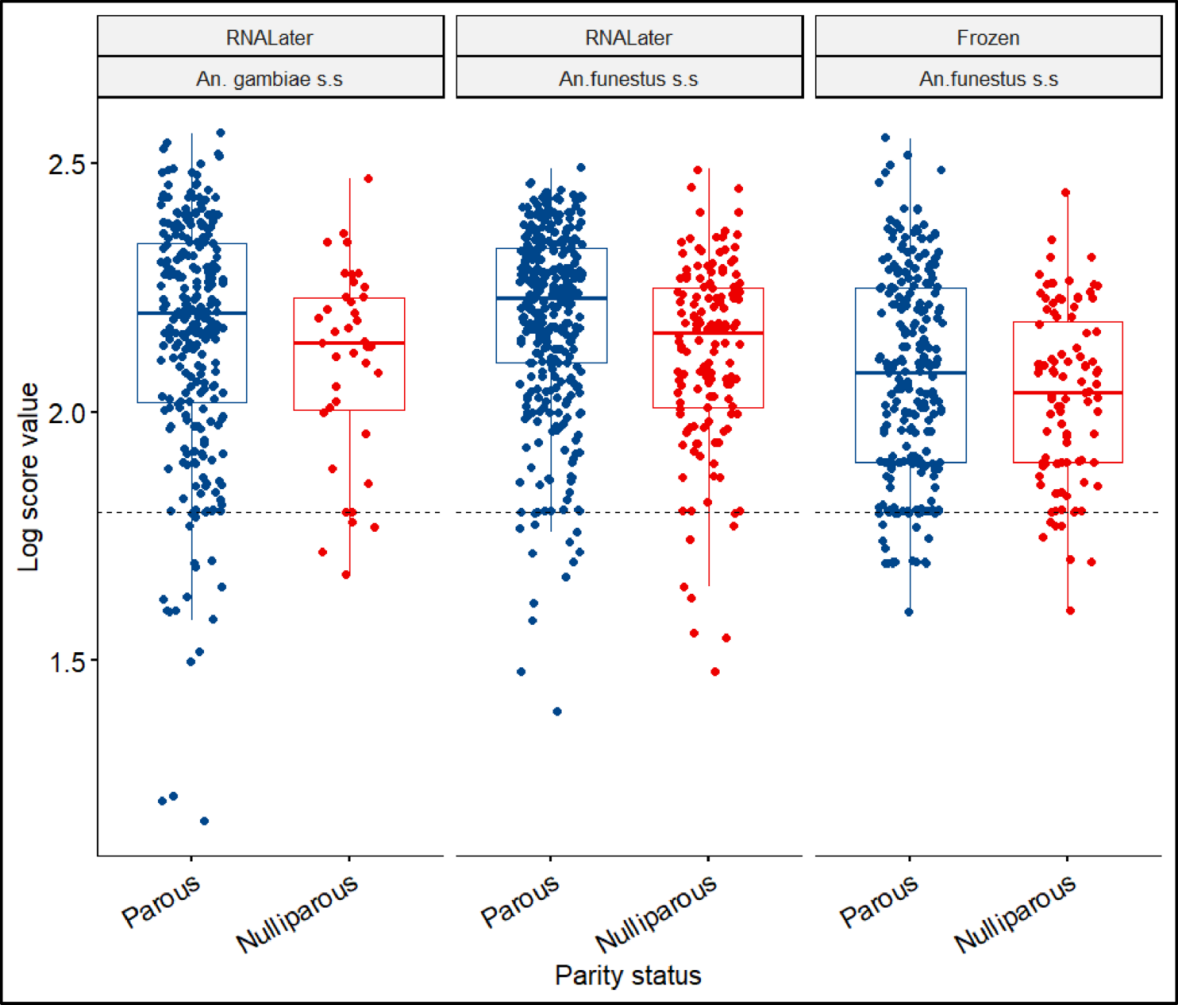
LSVs for the field collected *An. funestus s.s.* and *An. gambiae s.*s. from Kenya and Mozambique preserved under different storage conditions (RNALater and frozen at -20℃).

Samples stored in RNALater had an overall accuracy of 81% (95% CI:79, 84), while frozen samples had an accuracy of 87% (Table 3). For parity status determination, there was a fair agreement between MALDI-TOF MS and microscopy with Kappa values of 0.36 (95% CI: 0.28, 0.43) and 0.69 (95% CI: 0.6, 0.77) for both samples stored in RNALater and frozen respectively (Table 3). Sensitivity represents the capability of MALDI-TOF MS to correctly identify a truly parous mosquito as parous, and specificity as the ability to accurately identify a truly nulliparous mosquito as nulliparous. The area under the curve (AUC) for Mozambique samples (*An. funestus s.s.* and *An. gambiae* s.s.) stored in RNALater was 0.774%, and 0.695% respectively while that of Kenya samples (stored frozen) was 0.841% (Fig. 6). This shows a good performance by MALDI-TOF MS in distinguishing parous from nulliparous mosquitoes. Majority of LSV values for both complexes exceeded 1.8, indicating successful age categorization based on parity using MALDI-TOF MS. Nonetheless, a few cases had a LSV values below 1.8, likely attributed to protein degradation (Fig. 7).

## Discussion

The current study aimed at evaluating the utility of MALDI-TOF MS for age-grading of Afrotropical malaria vectors (insectary reared and wild-caught). We present evidence that the method can discern different mosquito age categories based on both physiological status and chronological age.

MALDI-TOF MS was able to correctly categorise insectary reared *An. gambiae* s.s. into; (i) Nulliparous blood-naïve; (ii) Nulliparous blood-wise; and (iii) Parous, with an overall accuracy of 94.52%. However, it was less accurate at differentiating between mosquitoes of multiple parous stages (first, second and third parous). This could be because although detection exploits protein changes those relating to oviposition events are likely similar across parous stages. Interestingly, the method was able to identify pre-gravid mosquitoes (a mosquito that has fed at least once in their life cycle). This finding can be used to evaluate accuracy of dissection methods which often underestimate survival due to high proportion of mosquitoes requiring two blood meals to lay eggs^30,31^. Compared to physiological status, the accuracy of chronological age prediction was much lower (77%). This suggests that although the ageing process may result in modification of mosquito protein profiles, these changes are likely to be less significant compared to those brought upon by physiological changes. Our findings demonstrate that protein changes are associated with age progression and correlate with key physiological milestones such as blood feeding, and oviposition. We hypothesize that these protein modifications can serve as reliable indicators for estimating the relative age of mosquitoes, given that wild mosquitoes are evolutionarily driven to undergo these physiological processes as part of their natural lifecycle. The exact nature of these protein alterations is yet to be established.

Equally, MALDI-TOF MS was successful in classifying wild-caught *An. funestus s.s* and *An. gambiae* s.s. mosquitoes by their parity status (overall accuracy 84%). However, storage conditions influenced the accuracy of the method, with frozen samples (-20℃) yielding a higher accuracy of 87%, compared to those stored in RNAlater™ (81%). Unfortunately, impact of sample storage in silica gel was not investigated in this study. Previous studies evaluating MALDI-TOF MS (for species prediction) have found that storage in silica gel is adequate to preserve protein profiles^23^, therefore future studies age grading could consider desiccants as a field-friendly storage alternative. A limitation of the study is that the reference method – Detinova dissection – may be prone to error depending on the technician and the integrity of the ovaries upon dissection^32^. However, no other alternative method is available for age grading hence the importance of this study. Quality control steps were taken during dissections to reduce potential error, and the two methods (dissection and MALDI-TOF MS) displayed a fair level of agreement.

Results from our insectary- based results indicate that MALDI-TOF MS databases for age-grading are likely best developed using reference spectra acquired from mosquitoes of different physiological ages. The development of such databases could be supported with data from wild caught samples to evaluate additional physiological states including mating, blood feeding and oviposition as described by Charlwood^30^. MALDI-TOF MS cost per sample processing is very low (< 0.2USD). The same spectrum which is collected for species identification can be used for age-grading provided libraries are available. The data analysis uses readily available software provided by Bruker making it very user friendly without the need for the end-user to be familiar with coding of deep learning methods. We were unable to determine whether a parity database generated from mosquitoes from one site can be used in a different site – this is because our storage conditions were different in Kenya and Mozambique and therefore not comparable. Techniques like NIRS and MIRS often observe a drop in cross site robustness requiring the development of site-specific databases^33,34^. However, cross site robustness is observed with MALDI-TOF MS for species identification, it is possible that the same applies to age-grading. The robustness of MALDI-TOF MS compared to other spectral methods may be explained by its focus on protein content rather than overall biochemical content. Further studies are needed to evaluate robustness of MALDI-TOF MS for age-grading across sites.

## Conclusion

Physiological changes in mosquito life cycle influence their MALDI-TOF MS protein profiles. These changes can be exploited to develop databases for age-grading. MALDI-TOF MS proved to have over 80% accuracy in predicting parity status (parous Vs nulliparous) of *Anopheles gambiae* s.s. and *Anopheles funestus* s.s. collected in Kenya and Mozambique. The same MALDI-TOF MS spectrum from mosquito cephalothorax can also be used to determine species and parity status, demonstrating the utility of the tool for multiple entomological assessments. However, research is needed to evaluate options for field friendly sample storage approaches and well as cross site robustness. Additionally, open-access MALDI TOF MS libraries are needed to enable dissemination of the technology.

## Methodology

We designed experiments to evaluate the ability of MALDI TOF MS to age grade Afro-tropical malaria vectors. In phase I, we evaluated the ability of MALDI TOF MS to predict physiological and chronological age in insectary-reared samples. In phase II, we used mosquitoes collected in the field to evaluate MALDI TOF MS ability to predict parity status, i.e. parous Vs nulliparous.

### Phase I – Evaluation of MALDI TOF MS ability to predict physiological and chronological age in insectary-reared samples

*Anopheles gambiae sensu stricto* (Kilifi strain) were reared to different physiological ages under normal insectary condition at a temperature of 26 ± 1 °C, and humidity of 80%, with 12 h light: 12 h dark cycle at KEMRI-Wellcome Trust Research Programme (KWRTP) insectary^35^. Upon pupation, 1800 pupae were harvested and separated into two cohorts – one cohort for physiological age and the other for chronological age assessment. For physiological age, 900 pupae were transferred into three different styrofoam paper cups, each cup placed in labelled cage and allowed to emerge. Two days post enclosion, 25 female adult mosquitoes obtained from each cage, labelled as potentially mated and nulliparous, killed by freezing at -20℃ and stored in individual microcentrifuge tubes. On the third day post enclosion, the remaining adult female mosquitoes were starved for 4 h and then fed on human blood. Twenty-four hours post blood feeding (PBF), 75 females were individually transferred into 1.5 ml microcentrifuge vials and frozen until further processing. The remaining *females* were placed individually in ovicups (small paper cups lined with a wet cotton, wet filter paper and covered with a net covering for egg laying). The mosquitoes were maintained on 10% glucose solution served on cotton pads. Following egg laying, 75 females were harvested and stored at – 20 °C awaiting processing (first parous group) while those that failed to oviposit (nulliparous/pregravid) were killed and stored. The rest of the females that had successfully oviposited and not harvested as part of the first parous batch were returned to the cages, blood fed for the second time, then returned into individual ovicups for a second oviposition event. Upon ovipositing, 75 females (second parous *group*) were harvested and stored as the previous cohorts. The remaining females were fed for a third time and a similar procedure as with the first and second parous groups followed.

For chronological age assessments, mosquitoes were reared to different ages without regard for their physiological age progression. This was done by transferring 900 pupae into three different cages and after enclosion maintaining adult mosquitoes using 10% glucose. On days 6 and 12 post enclosion, adult females were harvested and stored individually – 20 °C awaiting processing.

### Phase II – Evaluation of MALDI TOF MS ability to predict parity (parous Vs nulliparous) of wild caught mosquitoes

Adult mosquito sampling was done in Mopeia, Zambezi region of Mozambique and Kwale, Kenya. Sampling was done between 1700 h and 0700 h using CDC light traps set both indoors near sleeping areas and outdoors near livestock enclosures. Indoors, traps were hung on the foot side of the bed/sleeping area at approximately 1.5 m from the floor. Outdoors, traps were set up near livestock enclosures. Early the next morning (0700 h) traps were retrieved and transported to the laboratory for morphological identification to species complex level. Individual unfed anopheline females were dissected using the Detinova method^5^. Briefly, the female was held down by the anterior end of its abdomen using forceps. Using another pair of forceps, the 7th and 8th abdominal segments held and gently pulled to remove the ovaries^5^.The ovaries were placed on a drop of 1X PBS buffer solution on a microscope slide, teased apart, then examined under an optical microscope. The ovaries were categorized into either parous or nulliparous based on trachea filaments, and parity rates established. The remaining mosquito carcasses were stored individually in RNAlater or frozen then transported to KEMRI Wellcome Trust Research Programme (KWRTP) laboratory for further analysis.

#### Sample processing for MALDI-TOF MS spectra acquisition

*Sample cleaning and pre-processing*: Field collected mosquitoes stored in RNAlater were cleaned using ethanol then rinsed using distilled water before protein extraction. Frozen samples did not require cleaning and therefore proteins were extracted directly. The head and thorax were cut longitudinally into equal halves; one half for spectra acquisition with MALDI-TOF MS for species identification and database creation and validation for parity status. The other half for molecular analysis viz., species identification and *Plasmodium* screening. For the insectary-reared cohort, the entire cephalothorax was used for protein extractions.

*Protein extractions*: Acid glass beads wash was added, followed by addition of 15 µl of 50% acetonitrile and 15 µl of 70% formic acid. Samples were then lysed using TissueLyser II (Qiagen, Germany) machine (3 cycles of frequency 30 Hz for 1 min) followed by centrifugation at 15,000 rpm for 30 s. Thereafter, 2 µl of the supernatant was spotted on a MALDI-target (Bruker Daltonics) plate in quadruplicate, as previously described^23^. The MALDI plate was then air dried, samples overlaid with 2 µl matrix solution (α-cyano-4-hydroxycynnamic acid (Sigma-Aldrich, USA) 50% (v/v) acetonitrile, 2.5% (v/v) trifluoroacetic acid (Thermo scientific, USA) and 47.5% LC-MS grade water (Thermo scientific, USA)) and given time to dry at room temperature. The plate was then loaded into the MALDI-TOF MS machine for spectra acquisition^36^.

#### MALDI TOF MS spectra acquisition, processing, and database creation

Spectra were acquired using the FlexControl software ver. 3.3.0 (Bruker Daltonics) with a slight modification of 50% laser power and the rest of the parameters maintained at default setting as previously described^23^. The resulting spectra were then exported to FlexAnalysis software ver. 3.3.0 (Bruker Daltonics) for cleaning by checking their reproducibility, intensity and baseline smoothing, poor quality spectra and flat lines were removed. A sub-sample of the good quality spectra were randomly selected for parity status and age structure databases creation, while the remaining spectra were used for validation/querying the database. All good quality spectra were exported to MALDI Biotyper explorer software ver. 3.3.0 (Bruker Daltonics) for further spectra smoothing and normalization. Dendrograms were created from the main spectral profiles (MSPs) based on their age structures and parity status^23^.

*Age structure and parity status database creation and querying/validation*: Age structure and parity status database were created using MSPs using MALDI Biotyper explorer software ver. 3.3.0 (Bruker Daltonics). For each category, a minimum of 6 spectra per category were chosen for database creation. Spectra that had good reproducibility and intensity were loaded into MALDI Biotyper and further subjected to smoothing, checking for intensity, frequency, and peak picking position of the MSP spectra. Validation was performed on the remaining spectra by querying the unknown spectra of different ages with the reference spectra in the database. The MALDI Biotyper calculates a log score value (LSV) that ranges from 0 to 3, indicating the level of match between the unknown spectra and the reference database, LSV ≥ 1.8 was considered as reliable identification.

#### Molecular analysis of field-caught mosquitoes

*DNA extraction*: Half head and thorax were used for species identification and sporozoite screening. DNA extraction was done using Chelex method. In brief, Chelex solution was added into each sample, the tissue lysed, the heated for 10 min followed by centrifugation for 10 min. Supernatant for each sample was then transferred into a clean tube until further processing^37^.

*Species identification*: All field caught mosquitoes of *the An. funestus* and *An. gambiae* complex were identified to species using MALDI-TOF MS according to pre-published protocol^23^. Quality control by PCR was done on 10% of samples^38–40^. Equally, samples that generated poor spectra quality of Low LSVs upon species database query were also processed by PCR.

*Plasmodium screening*: Presence of *Plasmodium* infection in field caught mosquitoes was screened by PCR using 18s primers^41^.

## Data Availability

The supporting data is managed by the KEMRI-Wellcome Trust Data Governance Committee and can be accessed upon request directed to the committee. The dataset is available at: 10.7910/DVN/VJQXH3.
